# Potentially Toxic Elements in Costume Cosmetics Used by Children and Adults Are Associated with Cancer Risk

**DOI:** 10.3390/ijerph20010531

**Published:** 2022-12-28

**Authors:** Fernanda Junqueira Salles, Fernanda Pollo Paniz, Bruno Lemos Batista, Adelaide Cassia Nardocci, Kelly Polido Kaneshiro Olympio

**Affiliations:** 1 Department of Environmental Health, School of Public Health, University of Sao Paulo, Av. Dr. Arnaldo, 715, Cerqueira Cesar, São Paulo 01246-904, Brazil; 2 Center for Natural and Human Sciences, Federal University of ABC, Avenida dos Estados, 5001, Bairro Santa Terezinha, Santo André 9210-580, Brazil

**Keywords:** cosmetics, toxic elements, children exposure, occupational exposure, risk assessment

## Abstract

(1) Background: Costume cosmetics, such as face paints and pancakes, are used by adults and children during Halloween, Carnival, or children’s parties. However, the metallic-based pigments used as dyes in these products may contain toxic elements associated with different levels of exposure. Objectives: (a) to determine the Al, As, Ba, Cd, Co, Cr, Cu, Ni, Pb, Sb, Sn, and Sr concentrations in face paints and pancakes; and (b) to estimate cancer and non-cancer risks posed by the concentrations of each element in these products for dermal and ingestion exposure scenarios during children and adult use. (2) Methods: A total of 95 samples of face paints and pancakes (four brands in different textures and colors) were purchased at the largest high-street commercial center in São Paulo city, Brazil. An extraction procedure with nitric acid was carried out using a graphite-covered digester block. Toxic element determinations were performed using an ICP-MS. (3) Results: The non-cancer risks estimated were lower than 1, except for dermal exposure in adults for some target systems. High cancer risk values raise concerns in both groups. The risk for children ranged from 10^−8^ to 10^−5^ and proved higher in cases of accidental exposure by ingestion. For occupational exposure in adults, cancer risks were even higher, ranging from 10^−3^ to 10^−5^, with the highest values associated with dermal exposure. (4) Conclusions: The study results suggest the presence of potentially toxic elements (PTEs) in cosmetics should be regulated/monitored to protect human health, especially for occupational exposure and use by children.

## 1. Introduction

Every year the consumption of cosmetics increases worldwide. However, consumers may not completely understand the risks to their health associated when using these products [1]. Concern over the formulation of products has increased and this scrutiny has put pressure on the cosmetics industry [2,3]. Some metallic-based pigments used as dyes in face paints may contain toxic elements, such as heavy metals, raising doubts over their safety [4,5]. The use of these products may be associated with different levels of exposure, including via dermal and incidental ingestion routes.

The presence of toxic elements has already been detected and is well-documented in traditional cosmetic products such as lipsticks, eyeshadows, and skin creams [2,6,7,8,9]. However, few studies have investigated the presence of these elements in face paints used in costume makeup; the present study aims to close this knowledge gap. There is still some uncertainty regarding the tolerable values for the use of these products. In addition, exposure scenarios are difficult to assess and may vary depending on the cultural habits of each country. Wang et al. [5] found a high probability of developing cancer due to the lifetime exposure to high levels of heavy metals in face paints used by Chinese actors. Perez et al. [4] reported that costume cosmetics contain As, Co, Ni, Pb, and Sb which, in occupational exposures, may exceed health-based guidance values but did not pose a health risk to intermittent consumers in the user scenarios tested. In 2009, the Campaign for Safe Cosmetics in the USA found that all commercial face paints tested contained lead and 60% contained known skin allergens such as nickel, cobalt, and/or chromium at higher-than-recommended levels [10]. 

The face paints in liquid, cream, or pancake form are freshly applied to any part of the body, but most commonly to the head and trunk surfaces. These paints are used in occupational activities to convey a character’s personality and enhance the actor’s presence on stage [5]. However, they can also be used as costume cosmetics for adults and children. In the USA, this type of product is widely consumed during Halloween and is economically important during this festive season [4]. In Brazil, these paints are often used at children’s parties and during Carnival to complement the costumes of adults and children. Persistent contact with face paints can occur in long-term occupational exposure when the exposure levels to toxic elements can be higher in these users relative to the general population [5]. In children, even low levels of toxic elements can exert negative health effects, as the child and central nervous system are still developing [11]. Children are especially vulnerable to neurotoxic substances such as lead [11,12,13,14], whereas exposure to cadmium may have long-term consequences for bone composition and development [15]. In both cases, it is important to investigate the potential health risk to adults and children associated with the use of metal-containing face paints. Therefore, the aim of the present study was to help bridge this knowledge gap.

The objective of this study was to determine the concentration of twelve potentially toxic elements (PTEs: Al, As, Ba, Cd, Co, Cr, Cu, Ni, Pb, Sb, Sn, and Sr) in costume cosmetics (face paints and pancakes) available in high street outlets in São Paulo state, Brazil. Cancer and non-cancer risks posed by the content of each element in cosmetics were determined according to the models proposed by the United States Environmental Protection Agency (USEPA). The risks for face paints and pancakes were estimated considering exposure to the elements determined via dermal and non-dietary ingestion routes.

## 2. Materials and Methods

### 2.1. Samples

A total of 95 samples of face paints and pancakes were purchased from stores at the largest high street commercial center in the city of São Paulo, Brazil. The manufacturers of all the samples were Brazilian. Face paints of four brands in three different types (liquid, cream, and fluorescent) and multiple colors (red, yellow, black, white, green, orange, purple, blue, brown, pink, and lilac) were purchased (n = 90). All face paints available in the stores were purchased, whenever possible, in two different batches of each type and color. Pancakes for professional use were also evaluated, but only one brand was available for purchase in five different colors (blue, orange, red, yellow, and white; n = 5). 

### 2.2. Element Determinations

All samples were weighed out (150–200 mg in triplicate) and 2 mL of nitric acid (14 mol L^−1^) was added to each. Nitric acid digestion for metal determinations in cosmetic samples was reported previously by Lim et al. [9]. The resultant mixtures were kept overnight for pre-digestion to extract the elements (Al, As, Ba, Cd, Co, Cr, Cu, Ni, Pb, Sb, Sn, and Sr). Concentrated nitric acid (~65% m/m, Synth, Brazil) was sub-distilled before use (DST-1000, Savillex, USA). After pre-digestion, the heating procedure was carried out using a graphite-covered digester block (EasyDigest, Analab, France). The samples were heated at 120°C for 4 h, according to Paniz et al. [16The dermal cancer risk (*CR*) was calculated for As, Cr(VI), and Pb as shown in Equation (5), according to the USEPA [17].]. After cooling, the volume increased to 40 mL with deionized water (resistivity 18.2 MΩ·cm^−1^, Master System All, Gehaka, Brazil). Deionized water was used for all tests and cleaning. Before the ICP-MS analysis, the samples were filtered using a filter membrane of 0.2 µm (cellulose acetate).

Element determinations were performed using an inductively coupled plasma-mass spectrometer (ICP-MS, Agilent 7900, Hachioji, Japan). A multi-element standard solution was purchased from PerkinElmer with a concentration of 1000 µg L^−1^. For element determination by ICP-MS, an external calibration curve was prepared from standard multi-element solutions with concentrations of 1 µg L^−1^, 5 µg L^−1^, 10 µg L^−1^, 20 µg L^−1^, 50 µg L^−1^, 100 µg L^−1^, and 200 µg L^−1^. Blank solutions and certified reference materials (CRM) were also analyzed. To verify the accuracy of the procedure, CRM was prepared using the same procedure as for the samples. The CRM used were: NIST 2709 (San Joaquim soil), ERM CC 141 (Loam soil), NIST 1573 (Tomato Leaves), and Agro 1003a (Tomato Leaves). The ICP-MS operating conditions are shown in the Supplementary Materials (Table S1). The linearity of calibration lines was 1.00 for almost all elements analyzed, except for Pb which was 0.9999. The limits of detection (LODs) for elemental determination were calculated as three times the standard deviation of 10 independent measurements of the procedural blank (3σ criterion), divided by the slope of the calibration curve, and multiplied by the dilution factor. The LODs are presented in Table S1 of the Supplementary Materials. The recovery of elements from the CRMs analyzed is presented in Table S2 of the Supplementary Materials.

### 2.3. Dermal Exposure Assessment

During the application of face paints or pancakes to the skin, PTEs can undergo dermal absorption. The possibility of a person developing health problems or cancer due to the use and absorption of these products into the skin was evaluated by calculating the cancer risk (*CR*) and non-cancer risk or dermal hazard quotient (*HQ*) during the period of exposure.

The estimated dose by dermal absorption (*DAD*) was calculated according to Equations (1) and (2) for soil contact from USEPA [17].

(1) $${DA}_{event}=C\;\times \;CF\;\times \;AF\;\times \;ABS$$

(2) $$DAD=\frac{{DA}_{event}\;\times \;EF\;\times \;ED\;\times \;VV\;\times \;SA}{BW\;\times \;AT}$$

where *C* is the mean concentrations of PTE determined in the paints (mg kg^−1^); *CF* is a conversion factor defined by the USEPA as 10^−6^ mg kg^−1^ [17]; *AF* is the amount of skin adherence of the paints per event [5], obtained by dividing the average mass of paints applied to the skin per event by surface area (*SA*); *ABS* is a fraction of a specific metal absorbed dermally (As: 0.03; Cr(VI): 0.04; 0.001 for other PTEs) [5,18]; *DA_event_* is the dose absorbed per event (mg/cm^2^-event); *EF* is average exposure frequency considering hours per day and days per year [4]; *ED* is exposure duration in years [19]; *SA* is the average surface area that comes into contact with the paint, considering only the head surface for children and both head and trunk surfaces for adults [20]; *BW* is average body weight [21]; and *AT* is averaging time (carcinogenic risk, *AT* = 70 × 365 days; non-carcinogenic risk, *AT* = *ED* × 365 days). 

A list of all the variables and values used in the equations for dermal exposure is presented in Table 1.

The non-cancer risk or dermal hazard quotient (*HQ*) was calculated as shown in Equation (3) according to the USEPA [17].

(3) $$HQ=\frac{DAD}{RfD_{o}}\;\mathrm{or}\;HQ=\frac{DAD}{RfD_{abs}}$$

All the values used for *HQ* calculation are presented in Table 2. 

The *RfD_o_* is the oral reference dose (mg kg^−1^ day^−1^) from the USEPA/IRIS assessment and was used in *HQ* calculations for As and Sr. The Minimal Risk Levels (*MRL*) from the Agency for Toxic Substances and Disease Registry (ATSDR) were used for Al, Co, Cu, and Sn, whose *RfD_o_* were not available. In the absence of chronic *MRL*-oral values, intermediate *MRL*-oral values were used [22]. For Ba, Cd, Cr(III), Cr(VI), Ni, and Sb, the absorbed reference dose (*RfD_abs_*) was used instead of *RfD_o_*. The USEPA values were adopted only for elements with recommendations for adjustment of toxicity (Ba: 7; Cd: 5; CrIII: 1.3; CrVI: 2.5; Ni: 4; Sb: 15). The USEPA equation based on gastrointestinal absorbed dose was used to obtain the *RfD_abs_* (Equation (4)) [17].

(4) $$RfD_{abs}=RfD_{o}\times ABS_{gi}$$

where *ABS_gi_* is the fraction of contaminant absorbed in the gastrointestinal tract. 

The dermal cancer risk (*CR*) was calculated for As, Cr(VI), and Pb as shown in Equation (5), according to the USEPA [17].

(5) $$CR=DAD\times SF_{o}\;\mathrm{or}\;CR=DAD\times SF_{abs}$$

The *SF_o_* is the oral slope factor (mg kg^−1^ day^−1^) from the California Office of Environmental Health Hazard Assessment (OEHHA). This value was used in the equations for As and Pb. For Cr(VI), the USEPA equation based on gastrointestinal absorbed dose (Equation (6)) was used to obtain the absorbed cancer slope factor (*SF_abs_*) [17].

(6) $$SF_{abs}=\frac{SF_{o}}{ABS_{gi}}$$

### 2.4. Incidental Ingestion Exposure Assessment

Possible PTE exposure via incidental ingestion was investigated; therefore, the potential exposure from direct ingestion or ingestion via hand-to-mouth contact was evaluated. The exposure assessment was based on the statistical data provided in the USEPA Exposure Factors Handbook [20] and on calculations proposed by Perez et al. [4]. Hand-to-mouth contact values vary in the literature. The values used in the present study were similar to those observed and adopted by other studies [23].

The concentration of product applied per use was calculated using Equation (7), where *C* is the mean concentration determined for each PTE in the paint (mg kg^−1^), Mass is the number of grams of product applied per use [24], and *SA* is the surface area of the hand (cm^2^). The concentration of the product applied to the skin (*C_applied_*) can be used to calculate the oral intake (µg day^−1^) from hand-to-mouth contact (*Intake_HM_*) using Equation (8), where *SA_hand_* is the surface area of the hands [20]; *FSA_hand_* is the fractional surface area of the hand involved in hand-to-mouth contact [4]; λD is the hand-to-mouth frequency value in contacts per hour [25]; *f_D_* is the conversion factor for direct hand-to-mouth transfer efficiency (0.24) [26]; and *t* is the duration in hours per day that the cosmetic remains applied [4]. All variables and values used to calculate ingestion exposure are listed in Table 1.

(7) $$C_{applied}=\frac{C\times Mass}{SA}$$

(8) $$\;Intake_{HM}=C_{applied}\times SA_{hand}\times FSA_{hand}\times f_{D}\times \lambda_{D}\times t$$

Finally, dividing oral intake (µg day^−1^) by body weight (*BW*) yields oral dose (*D_oral_* in µg kg^−1^ day^−1^). This oral dose value was used to calculate ingestion cancer risk (*CR*) and hazard quotient (*HQ*). The same Equations (3) and (5) presented above were used, replacing the *DAD* value with the *D_oral_*, and using the *RfD_o_* and *SF_o_* for each element. 

### 2.5. Exposure Scenarios

Exposure to PTEs during the use of face paints and pancakes was assumed to occur through both dermal and incidental ingestion routes. Two exposure scenarios were considered: a child (age 2 to <11 years) who uses these products as costume cosmetics, and an adult (>21 years) in an occupational exposure scenario. For both of these situations, estimations were determined for the two biological sexes. Exposure was estimated using the mean concentrations of the detected PTEs. The estimates for children were determined for three different age groups (2 to <3, 3 to <6, and 6 to <11 years), with summed results representing risk during childhood (age 2 to <11 years).

For cumulative carcinogenic risks in case of exposure to multiple carcinogens, the risks of each substance were tallied. Information on non-additive interactions is not readily available and without this specific information, the cancer risk from various chemicals has been conservatively assumed to be additive [27]. Therefore, the carcinogenic risks (*CR*) were calculated as the sum of the As, Cr(VI), and Pb values determined, given their potential carcinogenic effects and the fact that carcinogenic slope factors were available for these elements, assuming a linear dose-response relationship [28]. The non-cancer risk or hazard quotient (*HQ*) was estimated for all elements except Pb. The final *HQ* values were summed by type of effect, i.e., according to the elements that had the same target system in the definition of the *RfD* or *MRL*, as shown in Table 2. This calculation was carried out according to Equations (9) and (10), where *LT* represents an averaging time equal to a mean lifetime of 70 years.

(9) $$CR=\sum DAD\times SF\times \frac{ED}{LT}$$

(10) $$HQ=\sum \frac{DAD}{RfD}\times \frac{ED}{LT}$$

For Cr(VI) risk estimates, the entire concentration of total chromium determined was considered hexavalent chromium. If the risk was within acceptable limits, this implied the lesser fraction of Cr(VI) would also be within safe limits, avoiding the need for chemical speciation [29,30]. A list of all variables and values used in the equations for dermal and oral exposure is presented in Table 1. The oral slope factor, minimal risk level (*MRL*), and reference doses (RfD) for the PTEs evaluated in this study are presented in Table 2.

### 2.6. Statistical Analysis

Descriptive statistical treatment of the PTE concentrations was performed, including, arithmetic mean, minimum and maximum, and the 95th percentiles of each element. 

The Kruskal–Wallis test was performed to evaluate the statistically significant differences in PTE concentrations among samples of different colors (red, yellow, black, white, green, orange, purple, blue, brown, pink, and lilac) and types (liquid, cream, fluorescent, and professional pancake). Dunn’s test of multiple comparisons was performed following a significant Kruskal–Wallis test (*p* < 0.05). All statistical analyses were conducted using R software [31].

**Table 1:** List of parameters used to assess dermal and oral exposure to PTEs from face paints and pancakes.

Variable	Description	Value	Unit	Reference
*DA_event_*	Dose absorbed per event	Varies by metal	mg cm^2^-event^−1^	
*C*	Element concentration	Varies by metal	mg kg^−1^	
*CF*	Conversion factor	1 × 10^−6^	mg kg^−1^	[17]
*AF*	Adherence factor to skin	Mass/SA	mg cm^2^-event^−1^	[5]
		Children: 196.08 ^a^; 166.67 ^b^; 151.52 ^c^ Adults: 260.42 ^d^; 207.68 ^e^		
Mass	Mass applied per application	Children: 1000 Adults: 20,000	mg	[4,24]
*SA*	Skin surface area	Children’s head surface: 510 ^a^; 600 ^b^; 660 ^c^ Adult’s head + trunk surface: 9630 ^d^; 7680 ^e^	cm^2^	[20]
*ABS*	Dermal absorption fraction	As: 0.03; Cr VI: 0.04; Other metals: 0.001	Unitless	[5,18]
*DAD*	Dermal absorbed dose	Varies by metal	mg kg^−1^ day^−1^	
*EF*	Exposure frequency	Children: 2 (4 h/day; 12 days/year)	days years^−1^	[4]
		Adults: 83 (8 h/day; 250 days/year)		
*ED*	Exposure duration	Children: 1 ^a^; 3 ^b^; 5 ^c^	years	[19]
		Adults: 35		
*EV*	Event frequency	1 event per day	events day^−1^	
*BW*	Body weight	Female children: 14.45 ^a^; 18.70 ^b^; 30.05 ^c^	kg	[21]
		Male children: 14.95 ^a^; 19.02 ^b^; 29.46 ^c^		
		Adults: 63.35 ^d^; 73.25 ^e^		
*AT*	Averaging time	*ED* × 365 days	days	[17]
*LT*	Lifetime	70	years	[17]
*C_applied_*	Concentration applied to the skin	Varies by metal	µg/cm^2^	
*SA_hand_*	Surface area of hands	Children: 280 ^a^; 370 ^b^; 510 ^c^	cm^2^	[20]
		Adults: 890 ^d^; 1070 ^e^		
*FSA_hand_*	Hand fractional surface area involved in hand-to-mouth contact	Children: 0.025 Adults: 0.0125	Unitless	[4]
*λ_D_*	Hand-to-mouth frequency	Children: 13 Adults: 8	Contacts/hour	[20,25]
*f_D_*	Conversion factor: direct hand-to-mouth transfer efficiency	0.24	Unitless	[26]
*t*	Time of oral exposure	Children: 4 Adults: 8	hours day^−1^	[4]
*Intake_HM_*	Oral intake from hand-to-mouth contact	Varies by metal	µg day^−1^	
*D_oral_*	Oral dose	Varies by metal	µg kg^−1^ day^−1^	

^a^ Values used for children aged from 2 to 3 years; ^b^ Values used for children aged from 3 to 6 years; ^c^ Values used for children aged from 6 to 11 years; ^d^ Values used for female adults; ^e^ Values used for male adults.

**Table 2:** Oral slope factor, minimal risk level (*MRL*), oral reference doses (*RfD_o_*), fraction of contaminant absorbed in the gastrointestinal tract (*ABS_gi_*), absorbed reference dose (*RfD_abs_*), and target systems considered for each element in the dermal and ingestion exposure assessment.

	Oral Slope Factor	*RfD_o_*	*MRL*	*ABS_gi_*	*RfD_abs_*	Target System
Al	-	-	1	-	-	Neurological
As	9.5	0.0003	-	-	-	Cardiovascular and dermal
Ba	-	0.2	-	7	1.4	Urinary
Cd	-	0.001	-	5	0.005	Urinary
Co	-	-	0.01	-	-	Hematological
Cr III	-	1	-	1,3	1.3	Other
CrVI	0.5	0.003	-	2,5	0.0075	Other
Cu	-	-	0.02	-	-	Gastrointestinal
Ni	-	0.02	-	4	0.08	Other
Pb	0.0085	-	-	-	-	-
Sb	-	0.0004	-	15	0.006	Hematological
Sn	-	-	0.3	-	-	Hematological
Sr	-	0.6	-	-	-	Musculoskeletal
Reference	OEHHA	USEPA/IRIS	ATSDR	USEPA		EPA/ATSDR
*RfD_o_* Oral Reference Dose						
*MRL* Minimal Risk Level						
*ABS_gi_* Fraction of contaminant absorbed in the gastrointestinal tract (dimensionless)						
*RfD_abs_* Absorbed reference dose (mg kg^−1^ day^−1^)						

## 3. Results

### 3.1. Element Concentrations in Samples

The face paints screened for the presence of PTEs were broken down into categories: face paints (liquid, cream, and fluorescent) and pancakes. Results that were below the limit of detection (<LOD: 18.7% for Cu, 9.4% for Cd, 6.3% for Sn) were assigned a value equal to the detection limit divided by the square root of 2 (LOD/√2) [32]. Results with relative standard deviation above 30% between triplicates were excluded from statistical tests and means (28% for Cu; 24% for Cd; 21.9% for Sn; 18.7 for Sb; 10.4% for Al; 3% for As, Sr, and Pb; 2% for Ba; and 1% for Co and Ni). The number of samples, mean, minimum and maximum values, and 95th percentile for each PTE determined are given in Table 3.

The PTEs that showed statistically significant differences between colors occurred with As, Ba, Cd, Co, Cu, and Pb. White, blue, and purple colors had the highest mean for Pb and Cd (*p* = 0.01). The highest As mean concentrations were found in lilac, brown, and white paints (*p* = 0.01). Lilac, blue, and green colors had the highest means for Cu (*p* < 0.01). The highest means for Ba were in red colors (*p* < 0.01), whereas for Co, the highest means were in brown and yellow (*p* = 0.03). For almost all elements determined (Al, As, Ba, Cd, Co, Cr, Ni, Pb, Sb, and Sn), the means were higher in the pancakes and liquid samples (*p* < 0.05). The cream samples and professional pancakes had higher means for Cd, Cr, and Pb (*p* < 0.0001). On the other hand, the means for Sr were higher in fluorescent and liquid paints. Only Cu did not differ in concentration between the types of costume cosmetics analyzed.

### 3.2. Cancer and Non-Cancer Risk

High cancer risk values raise concern, and arsenic was the element that contributed most to total risk (approximately 90%). In all exposure scenarios, the estimated cancer risks for the use of pancakes were higher than the risks for face paint consumption.

For children, dermal exposure risks exceeded 1 × 10^−8^ for face paints and 1 × 10^−7^ for pancakes. The accidental ingestion risk exceeded 1 × 10^−6^ for face paints and 1 × 10^−5^ for pancakes. For the general population, the tolerable acceptable risk is 1 × 10^−6^, whereas the USEPA deems a risk of 1 × 10^−4^ tolerable for specific and justified situations [33,34,35,36]. However, particularly in situations involving children, we considered a target of 1 × 10^−6^. In this case, the cancer risk values calculated for accidental ingestion by children exceeded this limit.

Dermal exposure values for adults exceeded 1 × 10^−3^ for pancakes and face paints, while ingestion risk exceeded 1 × 10^−5^ for face paints and 1 × 10^−4^ for pancakes in adults. The tolerable risk values in occupational exposure are variable. The National Institute for Occupational Safety and Health of the U.S. (NIOSH) considers a maximum of 1 × 10^−4^ [37]. By contrast, according to the European Commission [36], the risk for workers can vary from 1 × 10^−3^ to 1 × 10^−6^. Considering this variability and a tolerable range of 1 × 10^−4^ to 1 × 10^−5^, many values estimated in the present study proved high, with the highest values for dermal exposure in adults.

The non-cancer risks were lower than 1 for dermal exposure in children and ingestion exposure in both child and adult groups. Some values for dermal exposure in adults were greater than 1, where elements with the highest contribution to this total risk were arsenic and hexavalent chromium in pancakes. These reflect values greater than 1 for cardiovascular and dermal effects (100% As contribution) and other effects in pancakes (99% CrVI contribution).

None of the cancer and non-cancer risk values differed significantly between males and females. The total results for non-cancer (*HQ*) and cancer risk (*CR*) in child and adult exposure to face paints and pancakes are presented in Table 4. All the risk assessment results for each of the elements evaluated in this study are summarized in the Supplementary Materials (Tables S2 and S3).

## 4. Discussion

Elevated cancer risk values were found for both child and occupational exposures. In situations involving children, we adopted the target risk of 1 × 10^−6^ [35,36], while the tolerable range for adult workers was 1 × 10^−4^ to 1 × 10^−5^ [36,37]. The cancer risks for children during accidental exposure through ingestion proved higher than the risk due to dermal exposure. The specific child behavior of hand-to-mouth contact may contribute to relevant exposure for children [23]. This finding reinforces the importance of controlling the presence of these elements in products for children’s use. 

By contrast, the risk for adults was higher for dermal exposure, highlighting the importance of monitoring the presence of these elements in products for professional continuous use. Guidelines and limits for chemicals in products for professional consumption with a certified origin are also necessary. In this study, for example, the risks associated with pancakes of professional brands were higher than for face paints from the high street.

The exposure assessments used in this study were selected to conservatively estimate PTE exposures due to costume cosmetic application. As oral and dermal reference doses differ, toxicity factors (*ABS_gi_*) were applied based on EPA recommendations to account for the difference in absorbed dose relative to the administered dose and to avoid overestimation of risks. The EPA recommends adjustment for Ba, Cd, Cr, Ni, and Sb considering their absorption in the gastrointestinal system is low. For the other elements, the absorbed dose is equivalent to the administered dose, and therefore no toxicity adjustment was necessary [17].

The dermal and ingestion dose concentrations of toxic elements in this type of cosmetic are difficult to evaluate because of a lack of information regarding frequency and duration of use in adults and children, as well as the scarcity of data on the amount of costume cosmetic used per application [4]. Moreover, studies assessing health risks for cosmetics in adults are generally more common, whereas investigations evaluating the same risks in children are scarce. 

A few studies determining the concentrations of some elements in costume cosmetics have been conducted. Relative to the levels detected in the present study, Perez et al. [4] found a lower concentration of As and Cd (range for As: <0.079 to 0.53 mg kg^−1^; Cd < LOD) in costume eye-shadow and body paints sold in the United States, yet similar Co, Ni, and Pb concentrations (<0.5 to 2.0 mg kg^−1^; <0.20 to 6.3 mg kg^−1^; <0.15 to 9.3 mg kg^−1^, respectively) in pancakes; whereas Sb levels were higher in the US products (0.12–6.3 mg kg^−1^). The authors stated that the cumulative daily dose for all users did not exceed the *RfD* or *MRL* for As, Co, Ni, and Sb, and concluded that these concentrations do not pose a health risk to intermittent consumers and children, but occupational exposures may exceed health-based guidance values (1 × 10^−4^ mg kg^−1^ day^−1^). Wang et al. [5] assessed the health risks of face paint to Chinese opera actors. The mean concentrations of As, Cd, Co, Cu, Cr, Ni, and Pb detected in Chinese products were lower than those found in Brazil. For the total samples tested, *CR* ranged from 1.67 × 10^−7^ to 9.6 × 10^−3^. The carcinogenic risk in 25 paint samples ranged from 0.01% to 0.96%, with the highest risk for lifetime exposure to Cr-containing paints (above 1 × 10^−4^). 

Other studies have evaluated the health risks of different types of cosmetics (face makeup, eye shadow, and lipstick) for heavy metal contamination in products not specifically considered costume cosmetics. Lim et al. [9] found a hazard index of less than 1 for Al, Cr^3+^, Mn, Fe, Co, Ni, Cu, Zn, Cd, Sb, and Ti, but the cancer risk of dermal exposure to cosmetics in adults exceeded acceptable risk levels (>1 × 10^−5^). Arshad et al. [38] concluded that the cancer risk value was higher than the permissible limit in all cosmetic products tested (lotions, foundations, creams, hair dyes, and sunblock) except lipsticks. Ghaderpoori et al. [39] found the maximum value of oral cancer risk in creams (5.95 × 10^−6^) and the minimum value in eye pencils (5.29 × 10^−15^). Conversely, the hazard quotient, hazard index, and cancer risk were below acceptable limits in cosmetic products of different brands in Nigeria, indicating a measure of safety [40]. Kilic et al. [41] calculated the risk values for toxic metal concentrations in homemade cosmetic samples and found that all values were below 1, i.e., posed no health risk to humans. Another study found oral non-carcinogenic risk due to the Pb concentration in lipstick samples from Europe [42]. Samples of fairness creams, especially those with higher Hg levels, significantly exceeded the hazard quotient and hazard index tolerance limits [43].

Some countries have established regulations for the allowable amounts of heavy metals in cosmetics. According to Regulation (EC) No 1223/2009 of the European Union, heavy metals such as lead, cadmium, arsenic, and antimony were part of the list of substances prohibited in cosmetic products. However, the unintended presence of these metals in cosmetics is allowed if technically unavoidable [44]. In the regulation, there are no precise limits for these trace amounts, therefore, the German Federal Agency for Consumer Protection and Food Safety (BVL) issued a stringent standard for technically avoidable limits [45]. Of the face paint samples in the present study, 2.2% exceeded these limits for Pb (2 ppm), 2.7% for Sb (0.5 ppm), 10.9% for As (0.5 ppm), and 12.7% of the samples exceeded the limits for Cd (0.1 ppm). Health Canada established different limits for technically avoidable Pb (10 ppm), As (3 ppm), Cd (3 ppm), and Sb (5 ppm) [46]. Only 1.1% of the samples in the present study exceeded the Canadian limits for lead. The US Food and Drug Administration (FDA) allows a trace amount of less than 1 ppm of Hg and 10 ppm of lead in cosmetics and sets limits for color additives used, including 3 ppm of As, 20 ppm of Pb, and 1 ppm of Hg [47]. In Brazil, the MERCOSUR technical regulation is followed, which only stipulates a list of color additives permitted in cosmetics (20 ppm of Pb and 100 ppm for other heavy metals) [48]. Al-Saleh et al. [49] detected samples exceeding Pb limits, drawing attention to the need for a regular testing program to check for lead in cosmetics. Levels of arsenic exceeding the German standard for technically avoidable limits were also found in lipsticks, eye shadows, and eyebrow pencils [50]. The Environmental Defense of Canada also found make-up samples containing As levels above the national permissible limit [51]. Many countries have defined limits for cosmetic products based on levels that can be technically avoided but these are not based on risk assessment in which exposure conditions, such as amounts applied and duration of use, significantly influence the risks. The present study considered a conservative (but realistic) approach for an exposure scenario in Brazil, where children’s parties with face painting are common and some workers use paints frequently throughout the year. These conditions may vary by country based on different habits and local cultures.

Limits have been defined in legislation for only a few PTEs, while some countries have no established limits. An international agreement on the status and safety requirements of these products and their ingredients is needed [52]. Moreover, many specific products such as face paints do not have specific standards for metal concentrations. Even the most stringent EU regulations permit the non-intended presence of a small quantity of a prohibited substance, including heavy metals in finished cosmetic products, as technically unavoidable contaminations [1,2,41].

The available literature shows that different elements are present in many types of cosmetics produced worldwide [4,5,7,8,9,38,41,42,43,53]. The use of PTEs in these products is mainly due to their color properties [6]. Cosmetics with solid filler content, such as eye shadows, blushes, and compact powders, might contain more elemental contaminants than other cosmetic types [7]. The element content in cosmetics may act directly on the skin and cause allergic contact dermatitis, or be absorbed through the skin into the bloodstream, accumulating and exerting toxic effects in different organs. The risk of direct oral ingestion of cosmetics applied to the lips needs to be considered when licking lips or eating [2]. Cosmetics applied to the periocular area enable the ready absorption of elements into the blood because of the thinness of the skin in the region [54]. In addition, toxic elements may be absorbed through the conjunctiva and during lacrimation [2]. Additional information about the risks and possible health effects of each PTE evaluated in this study is presented in Appendix A.

Potentially toxic elements still exist in cosmetics. Therefore, it follows that the amounts applied to the skin or lips each day may accumulate over time. Moreover, countless cosmetics are on the market and may be used in combination, leading to different exposure patterns and health effects [6,38]. Notably, the risk values estimated in this study include only exposure to a specific type of cosmetics. However, a person’s lifetime exposure needs to take into account contributions from various exposure sources. Other hygiene and personal care products can also be a source of PTE exposure [52], adding to the risks estimated in the present study. Moreover, other sources unrelated to cosmetics may contribute to PTE exposure, such as toys, playground paints, diet, and occupational activities [55,56,57,58,59].

Special attention should be paid to the adverse health effects of cosmetic product consumption, considering the growing use, repeated exposure, and lack of uniform legislation governing the presence of toxic metals. It is paramount to investigate cumulative exposure and child use in a bid to improve draft guidelines on impurities in cosmetics and reflect technically avoidable contamination [1,51].

## 5. Conclusions

In conclusion, the data obtained in this study provides useful information regarding the content of PTEs in face paints and pancakes used as costume cosmetics in Brazil. Concerns about contaminated cosmetics are becoming commonplace in the beauty market, but limits for metal impurities in these products remain rare, and regulation governing levels are lacking in some countries. The non-cancer risks were lower than 1 for all exposure scenarios, except dermal exposure in adults for some target systems (in relation to definitions of target systems of oral RfD from As and CrVI). The cancer risk for children ranged from 10^−8^ to 10^−5^, proving higher in cases of accidental exposure by ingestion. For adults, cancer risks were even higher, ranging from 10^−3^ to 10^−5^, with the highest values associated with dermal exposure. The element contributing most to total risk values was arsenic (approximately 90%) and exposure scenarios for pancakes were associated with higher risk values.

These products are applied to children during parties, Halloween, and Carnival as part of the entertainment and celebrations. However, this exposure to chemicals at such a young age during these occasions, which are supposed to be safe and fun, is inappropriate and should be avoided. Further, this investigation of PTEs contained in costume cosmetic products suggests that the presence of these elements in cosmetics needs to be regulated and monitored in all countries to protect human health, especially regarding occupational exposure and child consumption.

## Figures and Tables

**Table 3:** The number of samples, arithmetic mean, minimum and maximum values, and 95th percentile for each Potentially Toxic Elements (PTEs) determined in face paints and pancakes.

PTEs mg/kg	Face Paints				Pancakes			
	n	Mean	Min–Max	95th Percentile	n	Mean	Min–Max	95th Percentile
Al	80	1420.71	5.91–19,325.04	9703.04	5	5082.81	490.49–20,717.28	20,717.28
As	87	0.19	0.01–1.69	0.76	5	0.43	0.08–1.24	1.24
Ba	88	173.42	0.70–33,700.51	1074.37	5	27.57	3.42–72.97	0.21
Cd	67	0.03	<0.01–0.25	0.12	4	0.21	0.01–0.40	0.40
Co	89	0.02	<0.01–0.15	0.05	5	0.46	0.04–1.01	1.01
Cr	90	0.64	0.09–5.32	2.58	5	12.15	1.04–22.63	22.63
Cu	63	67.77	<0.01–946.71	646.73	5	4.78	0.10–9.92	9.92
Ni	89	0.28	0.45–1.24	0.79	5	3.03	0.27–8.27	8.27
Pb	87	0.43	0.01–2.99	1.65	5	4.05	0.52–11.69	72.97
Sb	72	0.07	<0.01–2.65	0.24	4	0.02	<0.01–0.06	0.06
Sn	69	0.12	<0.01–1.11	0.38	5	0.26	<0.01–0.95	0.95
Sr	87	81.93	0.11–328.64	258.19	5	4.28	0.50–11.21	11.21

**Table 4:** Non-cancer (*HQ*) and cancer risk (*CR*) for dermal, ingestion, and total exposures for child and adult exposure to face paints and pancakes.

	Face Paints						Pancakes					
Child: 2 to < 11	Female			Male			Female			Male		
*HQ*	dermal	ingestion	total	dermal	ingestion	total	dermal	ingestion	total	dermal	ingestion	total
Hematological	4.20 × 10^−7^	3.18 × 10^−4^	3.18 × 10^−4^	4.18 × 10^−7^	3.16 × 10^−4^	3.17 × 10^−4^	1.49 × 10^−6^	1.63 × 10^−4^	1.65 × 10^−4^	1.48 × 10^−6^	1.62 × 10^−4^	1.64 × 10^−4^
Urinary	3.85 × 10^−6^	1.58 × 10^−3^	1.58 × 10^−3^	3.83 × 10^−6^	1.57 × 10^−3^	1.58 × 10^−3^	1.82 × 10^−6^	6.10 × 10^−4^	6.11 × 10^−4^	1.81 × 10^−6^	6.07 × 10^−4^	6.09 × 10^−4^
Gastrointestinal	1.01 × 10^−4^	5.73 × 10^−3^	5.83 × 10^−3^	1.00 × 10^−4^	5.70 × 10^−3^	5.80 × 10^−3^	7.11 × 10^−6^	4.22 × 10^−4^	4.29 × 10^−4^	7.08 × 10^−6^	4.20 × 10^−4^	4.27 × 10^−4^
Musculoskeletal	4.06 × 10^−6^	2.41 × 10^−4^	2.45 × 10^−4^	4.05 × 10^−6^	2.40 × 10^−4^	2.44 × 10^−4^	2.12 × 10^−7^	1.26 × 10^−5^	1.28 × 10^−5^	2.12 × 10^−7^	1.25 × 10^−5^	1.28 × 10^−5^
Cardiovascular, dermal	5.78 × 10^−4^	1.14 × 10^−3^	1.72 × 10^−3^	5.75 × 10^−4^	1.14 × 10^−3^	1.71 × 10^−3^	1.28 × 10^−3^	2.52 × 10^−3^	3.80 × 10^−3^	1.27 × 10^−3^	2.51 × 10^−3^	3.78 × 10^−3^
Neurological	4.23 × 10^−5^	2.51 × 10^−3^	2.55 × 10^−3^	4.21 × 10^−5^	2.50 × 10^−3^	2.54 × 10^−3^	1.51 × 10^−4^	8.97 × 10^−3^	9.12 × 10^−3^	1.51 × 10^−4^	8.93 × 10^−3^	9.08 × 10^−3^
Other	1.01 × 10^−4^	4.01 × 10^−4^	5.03 × 10^−4^	1.01 × 10^−4^	4.00 × 10^−4^	5.01 × 10^−4^	1.93 × 10^−3^	7.44 × 10^−3^	9.37 × 10^−3^	1.92 × 10^−3^	7.40 × 10^−3^	9.33 × 10^−3^
Cancer Risk	8.97 × 10^−8^	3.83 × 10^−6^	3.92 × 10^−6^	9.01 × 10^−8^	7.61 × 10^−6^	7.70 × 10^−6^	3.26 × 10^−7^	9.13 × 10^−5^	9.16 × 10^−5^	3.27 × 10^−7^	9.09 × 10^−5^	9.13 × 10^−5^
**Adults: ≥ 21**		**Female**			**Male**			**Female**			**Male**	
***HQ***	**dermal**	**ingestion**	**total**	**dermal**	**ingestion**	**total**	**dermal**	**ingestion**	**total**	**dermal**	**ingestion**	**total**
Hematological	5.03 × 10^−4^	5.46 × 10^−3^	5.96 × 10^−3^	4.35 × 10^−4^	4.72 × 10^−3^	5.15 × 10^−3^	1.78 × 10^−3^	2.80 × 10^−3^	4.58 × 10^−3^	1.54 × 10^−3^	2.42 × 10^−3^	3.96 × 10^−3^
Urinary	4.61 × 10^−3^	2.71 × 10^−2^	3.17 × 10^−2^	3.99 × 10^−3^	2.34 × 10^−2^	2.74 × 10^−2^	2.18 × 10^−3^	1.05 × 10^−2^	1.26 × 10^−2^	1.89 × 10^−3^	9.05 × 10^−3^	1.09 × 10^−2^
Gastrointestinal	1.21 × 10^−1^	9.83 × 10^−1^	2.19 × 10^−1^	1.05 × 10^−1^	8.50 × 10^−1^	1.90 × 10^−1^	8.53 × 10^−3^	7.24 × 10^−2^	1.58 × 10^−2^	7.37 × 10^−3^	6.26 × 10^−2^	1.36 × 10^−2^
Musculoskeletal	4.87 × 10^−3^	4.14 × 10^−3^	9.01 × 10^−3^	4.21 × 10^−3^	3.58 × 10^−3^	7.79 × 10^−3^	2.55 × 10^−4^	2.16 × 10^−4^	4.71 × 10^−4^	2.20 × 10^−4^	1.87 × 10^−4^	4.07 × 10^−4^
Cardiovascular, dermal	6.93 × 10^−1^	1.96 × 10^−2^	7.13 × 10^−1^	5.99 × 10^−1^	1.70 × 10^−2^	6.16 × 10^−1^	1.53 × 10^0^	4.33 × 10^−2^	1.57 × 10^0^	1.32 × 10^0^	3.75 × 10^−2^	1.36 × 10^0^
Neurological	5.07 × 10^−2^	4.31 × 10^−2^	9.37 × 10^−2^	4.38 × 10^−2^	3.72 × 10^−2^	8.11 × 10^−2^	1.81 × 10^−1^	1.54 × 10^−1^	3.35 × 10^−1^	1.57 × 10^−1^	1.33 × 10^−1^	2.90 × 10^−1^
Other	1.22 × 10^−1^	6.89 × 10^−3^	1.28 × 10^−1^	1.05 × 10^−1^	5.96 × 10^−3^	1.11 × 10^−1^	2.31 × 10^0^	1.28 × 10^−1^	2.44 × 10^0^	2.00 × 10^0^	1.10 × 10^−1^	2.11 × 10^0^
Cancer Risk	1.22 × 10^−3^	6.57 × 10^−5^	1.28 × 10^−3^	1.05 × 10^−3^	5.68 × 10^−5^	1.11 × 10^−3^	6.51 × 10^−3^	3.09 × 10^−4^	6.82 × 10^−3^	3.39 × 10^−3^	2.67 × 10^−4^	3.65 × 10^−3^

## Data Availability

The datasets generated and/or analyzed during the current study are available from the corresponding author on reasonable request.

## Supplementary Materials

The following supporting information can be downloaded at: https://www.mdpi.com/article/10.3390/ijerph20010531/s1, Table S1. Inductively Coupled Plasma-Mass Spectrometry operating conditions. Table S2. Element recoveries in percentage (%) compared to the certified value. Values are expressed as a median ± standard deviation of analyzed CRMs, n = 3. Table S3. Non-cancer risk or hazard quotient (*HQ*) for all Potentially Toxic Elements (PTEs) determined in face paints and pancakes, results for dermal and ingestion exposures for both sexes in children and adults. Table S4. Cancer risk (*CR*) for all Potentially Toxic Elements (PTEs) determined in face paints and pancakes, results for dermal and ingestion exposures for both sexes in children and adults.

## Appendix A

Of the elements determined in the present study, arsenic contributed the most to total risk values. The dermal uptake of arsenic is expected to be low, but when ingested, arsenic compounds are readily absorbed by the gastrointestinal tract and distributed throughout the body, accumulating predominantly in the liver, kidneys, lungs, spleen, and skin [60,61]. In chronic exposure, As will preferentially accumulate in tissues rich in keratin such as hair, nails, and skin. Adverse effects can include skin eruptions, but also skin cancer [61], classifying As as carcinogenic [62]. Long-term exposure via ingestion has also been associated with decreased blood cell production, blood vessel damage, foot and hand numbness, nausea, and diarrhea [60]. *HQ* values > 1 for cardiovascular and dermal as target organs were high in this study due directly to the concentration of arsenic in the samples since the oral RfD for arsenic defined this type of effect as the target.

Nickel is the most common contact allergen. At the epidermis level, Ni binds to amino acid residues forming an Ni-complexed protein that may cause a contact allergy, as well as irritation [6]. According to the International Agency for Research on Cancer [63], Ni compounds are carcinogenic to humans through inhalation exposure. Cobalt is also widely assumed to be a skin allergen, although few cases of Co-induced allergic reactions from cosmetic products have been described [6]. Co compounds are classified as possibly carcinogenic to humans [64]. Chromium oxidation states also can lead to the development of a contact allergy. Due to the higher solubility of Cr(VI), this type permeates the skin more than Cr(III) [6]. The IARC has classified Cr(VI)compounds as carcinogenic to humans but not Cr(III). The cancer risk for Cr(VI) in this study ranged from 10^-3^ in adults to 10^-8^ in children and, in some exposure scenarios, *HQ* values were >1 (Tables S3 and S4 in the Supplementary Material). Given the exposure assessment assumes all Cr present is in the form of Cr(VI), it may be necessary to perform chemical speciation of the Cr components [29].

Pb compounds are prohibited in most cosmetics, but impurities can be found in raw materials or acquired during the manufacturing process [6]. Inorganic Pb compounds are classified as probably carcinogenic [65]. Principal exposure routes are ingestion or inhalation, but dermal absorption has also been reported [6]. The US Centers for Disease Control and Prevention (CDC) stated that no safe level in blood can be established [66], with even the lowest levels having been shown to affect the fetus and central nervous system in children [11,12,14]. Cadmium tends to accumulate in the kidneys and liver regardless of the exposure route [67]. Chronic exposure to low levels of Cd can also cause bones to become brittle and prone to fracture. Dermal absorption is not a significant route of Cd entry as ingestion is more significant [6]. Cd is classified as carcinogenic to humans [68]. Dermal absorption of Sb has not been well studied, but Sb ingestion can cause gastrointestinal effects, including abdominal pain, vomiting, diarrhea, and ulcers [69]. Only Sb trioxides are classified as possibly carcinogenic to humans [70].

Aluminum, barium, tin, copper, and strontium are not on IARC’s list of carcinogens. Dermal contact for these elements is probably a minor route of exposure, while the primary route is oral [71,72,73,74,75]. Exposure to high levels of Al may cause neurological and skeletal effects in adults and children [75]. Scant human and animal data are available for Ba, Sn, Cu, and Sr. In general, dermal or oral exposure to these elements leads to gastrointestinal effects [61,63,74]. In addition, problems with bone growth may occur in children after high levels of Sr exposure [72].

## References

[1] Marinovich M., Boraso M.S., Testai E., Galli C.L. (2014). Metals in cosmetics: An a posteriori safety evaluation. Regul. Toxicol. Pharmacol..

[2] Borowska S., Brzóska M.M. (2015). Metals in cosmetics: Implications for human health. J. Appl. Toxicol..

[3] Tsatalis J.P., Aldahan A.S., Hsu V.M., Tsatalis A.E., Brah T.K., Nouri K. (2017). Narcissus’ reflection: Toxic ingredients in cosmetics through the ages. Int. J. Dermatol..

[4] Perez A.L., Nembhard M., Monnot A., Bator D., Madonick E., Gaffney S.H. (2017). Child and adult exposure and health risk evaluation following the use of metal- and metalloid-containing costume cosmetics sold in the United States. Regul. Toxicol. Pharmacol..

[5] Wang B., Su Y., Tian L., Peng S., Ji R. (2020). Heavy metals in face paints: Assessment of the health risks to Chinese opera actors. Sci. Total Environ..

[6] Bocca B., Pino A., Alimonti A., Forte G. (2014). Toxic metals contained in cosmetics: A status report. Regul. Toxicol. Pharmacol..

[7] Hepp N.M., Mindak W.R., Gasper J.W., Thompson C.B., Barrows J.N. (2014). Survey of cosmetics for arsenic, cadmium, chromium, cobalt, lead, mercury, and nickel content. J. Cosmet. Sci..

[8] Aldayel O., Hefne J., Alharbi K.N., Al-Ajyan T. (2018). Heavy Metals Concentration in Facial Cosmetics. Nat. Prod. Chem. Res..

[9] Lim D.S., Roh T.H., Kim M.K., Kwon Y.C., Choi S.M., Kwack S.J., Kim K.B., Yoon S., Kim H.S., Lee B. (2018). Non-cancer, cancer, and dermal sensitization risk assessment of heavy metals in cosmetics. J. Toxicol. Environ. Health. Part A.

[10] Sarantis H., Malkan S., Archer L. (2009). In Pretty Scary: Could Halloween Face Paint Cause Lifelong Health Problems. https://womensvoices.org/wp-content/uploads/2010/06/PrettyScary_FinalRpt_Oct2009.pdf.

[11] Dórea J.G. (2019). Environmental exposure to low-level lead (Pb) co-occurring with other neurotoxicants in early life and neurodevelopment of children. Environ. Res..

[12] Olympio K.P.K., Gonçalves C., Günther W.M.R., Bechara E.J.H. (2009). Neurotoxicity and aggressiveness triggered by low-level lead in children: A review. Rev. Panam. Salud Pública/Pan Am. J. Public Health..

[13] Olympio K.P., Oliveira P.V., Naozuka J., Cardoso M.R., Marques A.F., Günther W.M., Bechara E.J. (2010). Surface dental enamel lead levels and antisocial behavior in Brazilian adolescents. Neurotoxicol. Teratol..

[14] Olympio K.P.K., Goncalves C.G., Salles F.J., Ferreira A.P.S.S., Silva A.S., Buzalaf M.A.R., Cardoso M.R.A., Bechara E.J.H. (2017). What are the blood lead levels of children living in Latin America and the Caribbean?. Environ. Int..

[15] Waseem A., Arshad J. (2016). A review of human biomonitoring studies of trace elements in Pakistan. Chemosphere.

[16] Paniz F.P., Pedron T., Freire B.M., Torres D.P., Silva F.F., Batista B.L. (2018). Effective procedures for the determination of As, Cd, Cu, Fe, Hg, Mg, Mn, Ni, Pb, Se, Th, Zn, U and rare earth elements in plants and foodstuffs. Anal. Methods..

[17] USEPA, United States Environmental Protection Agency (2004). Risk Assessment Guidance for Superfund Volume I: Human Health Evaluation Manual (Part E). https://www.epa.gov/sites/production/files/2015-09/documents/part_e_final_revision_10-03-07.pdf.

[18] Chaparro Leal L.T., Guney M., Zagury G.J. (2018). In vitro dermal bioaccessibility of selected metals in contaminated soil and mine tailings and human health risk characterization. Chemosphere.

[19] Brazil. Constitution Text of 5 October 1988, Article 7. https://www.refworld.org/docid/4c4820bf2.html.

[20] USEPA, United States Environmental Protection Agency (2011). Exposure Factors Handbook.

[21] IBGE, Instituto Brasileiro de Geografia e Estatística (2010). Pesquisa de Orçamentos Familiares 2008–2009: Antropometria e Estado Nutricional de Crianças, Adolescentes e Adultos no Brasil. Tabela 1.1-Dados Amostrais e Estimativas Populacionais das Medianas de Altura e peso, por Situação do Domicílio e Sexo, Segundo a Idade e os Grupos de Idade Brasil-Período 2008–2009.

[22] ATSDR, Agency for Toxic Substances and Disease Registry Minimal Risk Levels (MRLs)—For Professionals.

[23] ter Burg W., Bremmer H.J., van Engelen J.G.M. (2008). Oral Exposure of Children to Chemicals via Hand-to-Mouth Contact.

[24] Loretz L., Api A., Barraj L., Burdick J., Dressler W., Gettings S., Hsu H.H., Pan Y., Re T., Renskers K. (2005). Exposure data for cosmetic products: Lipstick, body lotion, and face cream. Food Chem. Toxicol..

[25] Nicas M., Best D. (2008). A study quantifying the hand-to-face contact rate and its potential application to predicting respiratory tract infection. J. Occup. Environ. Hyg..

[26] Sahmel J., Hsu E.I., Avens H.J., Beckett E.M., Devlin K.D. (2015). Estimation of hand to-mouth transfer efficiency of lead. Ann. Occup. Hyg..

[27] USEPA, United States Environmental Protection Agency (2018). Technical Support Document United States Environmental Protection Agency. EPA’s 2014 National Air Toxics Assessment. https://www.epa.gov/sites/production/files/2018-09/documents/2014_nata_technical_support_document.pdf.

[28] USEPA, United States Environmental Protection Agency (2005). Guidelines for Carcinogen Risk Assessment.

[29] Doyi I.N.Y., Isley C.F., Soltani N.S., Taylor M.P. (2019). Human exposure and risk associated with element concentrations in indoor dust from Australian homes. Environ. Int..

[30] Maseki J., Annegarn H.J., Spiers G. (2017). Health risk posed by enriched heavy metals (As, Cd, and Cr) in airborne particles from Witwatersrand gold tailings. J. S. Afr. Inst. Min. Metall..

[31] R Core Team (2020). R: A Language and Environment for Statistical Computing.

[32] Croghan C., Egeghy P.P. (2003). Methods of Dealing with Values Below the Limit of Detection Using SAS. Presented at Southeastern SAS User Group. https://cfpub.epa.gov/si/si_public_record_report.cfm?Lab=NERL&dirEntryId=64046.

[33] USEPA, United States Environmental Protection Agency (1991). Environmental Risk: Your Guide to Analyzing and Reducing Risk.

[34] CFR 40 Code of Federal Regulations. Section 300.430-Remedial Investigation/Feasibility Study and Selection of Remedy (e)(2)(I)(A)(2)..

[35] Health Canada (2013). Federal Contaminated Sites Risk Assessment in Canada, Interim Guidance on Human Health Risk Assessment for Short-Term Exposure to Carcinogens at Contaminated Sites. www.hc-sc.gc.ca/ewh-semt/contamsite/index-eng.php.

[36] EC, European Commission (2016). Internal Market, Industry, Entrepreneurship and SMEs: Workshop on ‘Acceptable Level of Risk to Workers and Consumers Exposed to Carcinogenic Substances’.

[37] Whittaker C., Rice F., McKernan L., Dankovic D., Lentz T.J., MacMahon K., Kuempel E., Zumwalde R., Schulte P. (2016). Current Intelligence Bulletin 68: NIOSH Chemical Carcinogen Policy.

[38] Arshad H., Mehmood M.Z., Shah M.H., Abbasi A.M. (2020). Evaluation of heavy metals in cosmetic products and their health risk assessment. Saudi Pharm. J..

[39] Ghaderpoori M., Kamarehie B., Jafari A., Alinejad A.A., Hashempour Y., Saghi M.H., Ferrante M. (2020). Health risk assessment of heavy metals in cosmetic products sold in Iran: The Monte Carlo simulation. Environ. Sci. Pollut. Res..

[40] Ajaezi G.C., Amadi C.N., Ekhator O.C., Igbiri S., Orisakwe O.E. (2018). Cosmetic Use in Nigeria May Be Safe: A Human Health Risk Assessment of Metals and Metalloids in Some Common Brands. J. Cosmet. Sci..

[41] Kilic S., Kilic M., Soylak M. (2021). The Determination of Toxic Metals in some Traditional Cosmetic Products and Health Risk Assessment. Biol. Trace Elem. Res..

[42] Lara-Torres S., Figueiredo D., Paz S., Gutiérrez A.J., Rubio C., González-Weller D., Revert C., Hardisson A. (2021). Determination and risk assessment of toxic metals in lipsticks from Europe and China. J. Trace Elem. Med. Biol..

[43] Irfan M., Shafeeq A., Siddiq U., Bashir F., Ahmad T., Athar M., Butt M.T., Ullah S., Mukhtar A., Hussien M. (2022). A mechanistic approach for toxicity and risk assessment of heavy metals, hydroquinone and microorganisms in cosmetic creams. J. Hazard Mater..

[44] EU, European Union (2009). Regulation (EC) No 1223/2009 of the European Parliament and of the Council of 30 November 2009 on cosmetic products. Off. J. Eur. Union L.

[45] Bund B. (2017). Technically avoidable heavy metal contents in cosmetic products. J Consum. Prot. Food Saf..

[46] Health Canada (2012). Guidance on Heavy Metal Impurities in Cosmetics, vol. 2013. Consumer Product Safety. http://www.hc-sc.gc.ca/cps-spc/pubs/indust/heavy_metals-metaux_lourds/index-eng.php.

[47] US FDA, 21 CFR Part 700. Food and Drug Administration, Department of Health and Human Services. Subchapter G: Cosmetics. https://www.ecfr.gov/current/title-21/chapter-I/subchapter-G/part-700?toc=1.

[48] Brasil, 2012 [In Portuguese]. Resolução-RDC Nº 44, de 9 de Agosto de 2012. National Health Surveillance Agency. Health Ministry. https://bvsms.saude.gov.br/bvs/saudelegis/anvisa/2012/rdc0044_09_08_2012.html.

[49] Al-Saleh I., Al-Enazi S., Shinwari N. (2009). Assessment of lead in cosmetic products. Regul. Toxicol. Pharm..

[50] Saadatzadeh A., Afzalan S., Zadehdabagh R., Tishezan L., Najafi N., Seyedtabib M., Noori S.M.A. (2019). Determination of heavy metals (lead, cadmium, arsenic, and mercury) in authorized and unauthorized cosmetics. Cutan. Ocul. Toxicol..

[51] ED, Environmental Defence Canada (2011). Heavy Metal Hazard. The Health Risks of Hidden Heavy Metals in Face Makeup.

[52] Nohynek J.G., Antignac E., Re T., Toutain H. (2010). Safety assessment of personal care products/cosmetics and their ingredients. Toxicol. Appl. Pharmacol..

[53] Chen K.L., Jiang S.J., Chen Y.L. (2017). Determining lead, cadmium and mercury in cosmetics using sweeping via dynamic chelation by capillary electrophoresis. Anal Bioanal. Chem..

[54] Pratchyapruit W., Kikuchi K., Gritiyarangasan P., Aiba S., Tagami H. (2007). Functional analyses of the eyelid skin constituting the most soft and smooth area on the face: Contribution of its remarkably large superficial corneocytes to effective water-holding capacity of the stratum corneum. Skin Res. Technol..

[55] Leroux I.N., Ferreira A.P.S.D.S., Paniz F.P., Pedron T., Salles F.J., da Silva F.F., Maltez H.F., Batista B.L., Olympio K.P.K. (2018). Lead, Cadmium, and Arsenic Bioaccessibility of 24 h Duplicate Diet Ingested by Preschool Children Attending Day Care Centers in Brazil. Int. J. Env. Res. Public Health.

[56] Leroux I.N., Ferreira A.P.S.D.S., Silva J.P.D.R., Bezerra F.F., da Silva F.F., Salles F.J., Luz M.S., de Assunção N.A., Cardoso M.R.A., Olympio K.P.K. (2018). Lead exposure from households and school settings: Influence of diet on blood lead levels. Environ. Sci. Pollut. Res. Int..

[57] Silva J.P.R., Salles F.J., Leroux I.N., da Silva Ferreira A.P.S., da Silva A.S., Assunção N.A., Nardocci A.C., Sayuri Sato A.P., Barbosa F., Cardoso M.R.A. (2018). High blood lead levels are associated with lead concentrations in households and day care centers attended by Brazilian preschool children. Environ. Pollut..

[58] Ferreira A.P.S.S., Pereira E.C., Salles F.J., Silva F.F., Batista B.L., Handakas E., Olympio K.P.K. (2019). Home-based and informal work exposes the families to high levels of potentially toxic elements. Chemosphere.

[59] Salles F.J., Tavares D.J.B., Freire B.M., Ferreira A.P.S.S., Handakas E., Batista B.L., Olympio K.P.K. (2021). Home-based informal jewelry production increases exposure of working families to cadmium. Sci. Total Environ..

[60] ATSDR, Agency for Toxic Substances and Disease Registry (2007). Toxicological Profile for Arsenic.

[61] Health Canada (2010). Report on Human Biomonitoring of Environmental Chemicals in Canada: Results of the Canadian Health Measures Survey Cycle 1 (2007–2009).

[62] IARC, International Agency for Research on Cancer (2012). Arsenic, Metals, Fibres, and Dusts.

[63] IARC, International Agency for Research on Cancer (2012). Nickel and Nickel Compounds.

[64] IARC, International Agency for Research on Cancer (1991). Chlorinated Drinking-Water; Chlorination By-Products; Some Other Halogenated Compounds; Cobalt and Cobalt Compounds.

[65] IARC, International Agency for Research on Cancer (2006). Inorganic and Organic Lead Compounds.

[66] CDC, Center for Disease Control and Prevention (2012). Low Level Lead Exposure Harms Children: A Renewed Call for Primary Prevention. Report of the Advisory Committee on Childhood Lead Poisoning Prevention.

[67] ATSDR, Agency for Toxic Substances and Disease Registry (2012). Toxicological Profile for Cadmium.

[68] IARC, International Agency for Research on Cancer (2012). Cadmium and Cadmium Compounds.

[69] ATSDR, Agency for Toxic Substances and Disease Registry (1992). Toxicological Profile for Antimony.

[70] IARC, International Agency for Research on Cancer (1989). Some Organic Solvents, Resin Monomers and Related Compounds, Pigments and Occupational Exposures in Paint Manufacture and Painting.

[71] ATSDR, Agency for Toxic Substances and Disease Registry (2004). Toxicological Profile for Copper.

[72] ATSDR, Agency for Toxic Substances and Disease Registry (2004). Toxicological Profile for Strontium.

[73] ATSDR, Agency for Toxic Substances and Disease Registry (2005). Toxicological Profile for Tin.

[74] ATSDR, Agency for Toxic Substances and Disease Registry (2007). Toxicological Profile for Barium.

[75] ATSDR, Agency for Toxic Substances and Disease Registry (2008). Toxicological Profile for Aluminum.

