# Supplementary material for: Potentially Toxic Elements in Costume Cosmetics Used by Children and Adults Are Associated with Cancer Risk
Source: Int J Environ Res Public Health. 2022 Dec 28;20(1):531. doi: 10.3390/ijerph20010531 (PMC9819701; doi:10.3390/ijerph20010531)
Supplement: Supplementary file 1 [file ijerph-20-00531-s001.zip › ijerph-2065093-supplementary.pdf]

Supplementary Material

Table S1. Inductively Coupled Plasma-Mass Spectrometry operating conditions

Operating conditions for ICP-MS	
Isotopes monitored and respective limit of detection (µg L^{-1})	*^{27}Al(3.41), ^{52}Cr(0.047), ^{59}Co(0.013), ^{60}Ni(0.432), ^{63}Cu(2.92), ^{75}As(0.034),^{88}Sr(0.049), ^{111}Cd(0.006), ^{120}Sn(0.063), ^{121}Sb(0.013), ^{137}Ba(0.074), ^{208}Pb(0.045).*
Internal Standards	Ge^{72} (used for Al, Cr, Co, Ni and Cu)<br>Y^{89} (used for As, Sr, Cd, Sn and Sb)<br>Ir^{193} (used for Ba and Pb)<br>Purchased from Inorganic Ventures (USA). Prepared at 10 µg L^{-1} and injected online by splitting.
Radio Frequency Power	1550 W
Torch sample depth	8 mm
Argon Flow Rate	15 L min^{-1}
Nebulizer Gas Flow Rate	0.9 L min^{-1}
Nebulizer	Micro Mist™
Peristaltic Pump tuning	Internal diameter: 1.02mm; wall 0.86mm
Sample uptake	0.45 rps
Sample introduction	1 ml/min (0.3 rps)
Carrier Nebulizer Gas Flow rate	1.03 L min^{-1}
Stabilization time	5 s
Collision Cell	Helium (purity >99.99%)
Nebulizer Chamber	Scott (double pass)
Interface	Platinum cones
Sampling Cone	1 mm
Skimmer	0.9 mm

Table S2. Element recoveries in percentage (%) compared to the certified value. Values are expressed as a median ± standard deviation of analyzed CRMs, n=3.

Element	CRM	Recovery % (median ± SD)
Al	NIST 2709	76 ± 7
Cr	NIST 2709	111 ± 2
Co	NIST 2709	88 ± 2
Ni	NIST 2709	82 ± 2
Cu	NIST 1573	88 ± 1
As	C1003a	106 ± 2
Sr	C1003a	88 ± 2
Cd	CC141	94 ± 1
Sn	NI	-
Sb	NIST 1573	92 ± 7
Ba	NIST 2709	84 ± 6
Pb	CC141	90 ± 1

NI: not informed

Table S3. Non-cancer risk or hazard quotient (HQ) for all Potentially Toxic Elements (PTEs) determined in face paints and pancakes, results for dermal and ingestion exposures for both sexes in children and adults.

HQ		Children: age 2 to < 3 years				Children: age 3 to < 6 years				Children: age 6 to < 11 years				Adults: age ≥ 21 years			
		Face paints		Pancake		Face paints		Pancake		Face paints		Pancake		Face paints		Pancake	
PTEs	Sex	Dermal	Ingestion	Dermal	Ingestion	Dermal	Ingestion	Dermal	Ingestion	Dermal	Ingestion	Dermal	Ingestion	Dermal	Ingestion	Dermal	Ingestion
**Al**	F	5.17×10^{-4}	3.07×10^{-2}	1.85×10^{-3}	1.10×10^{-1}	4.00×10^{-4}	2.37×10^{-2}	1.43×10^{-3}	8.48×10^{-2}	2.49×10^{-4}	1.48×10^{-2}	8.90×10^{-4}	5.28×10^{-2}	1.01×10^{-1}	8.61×10^{-2}	3.63×10^{-1}	3.08×10^{-1}
	M	5.00×10^{-4}	2.96×10^{-2}	1.79×10^{-3}	1.06×10^{-1}	3.93×10^{-4}	2.33×10^{-2}	1.41×10^{-3}	8.34×10^{-2}	2.54×10^{-4}	1.50×10^{-2}	9.08×10^{-4}	5.38×10^{-2}	8.77×10^{-2}	7.45×10^{-2}	3.14×10^{-1}	2.66×10^{-1}
**As**	F	7.07×10^{-3}	1.40×10^{-2}	1.56×10^{-2}	3.09×10^{-2}	5.46×10^{-3}	1.08×10^{-2}	1.21×10^{-2}	2.38×10^{-2}	3.40×10^{-3}	6.72×10^{-3}	7.50×10^{-3}	1.48×10^{-2}	1.39×10^{0}	3.92×10^{-2}	3.06×10^{0}	8.66×10^{-2}
	M	6.83×10^{-3}	1.35×10^{-2}	1.51×10^{-2}	2.98×10^{-2}	5.37×10^{-3}	1.06×10^{-2}	1.19×10^{-2}	2.34×10^{-2}	3.47×10^{-3}	6.86×10^{-3}	7.65×10^{-3}	1.51×10^{-2}	1.20×10^{0}	3.39×10^{-2}	2.65×10^{0}	7.49×10^{-2}
**Ba**	F	4.51×10^{-5}	1.87×10^{-2}	7.17×10^{-6}	2.98×10^{-3}	3.48×10^{-5}	1.45×10^{-2}	5.54×10^{-6}	2.30×10^{-3}	2.17×10^{-5}	9.00×10^{-3}	3.45×10^{-6}	1.43×10^{-3}	8.84×10^{-3}	5.26×10^{-2}	1.41×10^{-3}	8.36×10^{-3}
	M	4.36×10^{-5}	1.81×10^{-2}	6.93×10^{-6}	2.88×10^{-3}	3.43×10^{-5}	1.42×10^{-2}	5.45×10^{-6}	2.26×10^{-3}	2.21×10^{-5}	9.18×10^{-3}	3.52×10^{-6}	1.46×10^{-3}	7.64×10^{-3}	4.55×10^{-2}	1.22×10^{-3}	7.23×10^{-3}
**Cd**	F	1.96×10^{-6}	5.82×10^{-4}	1.51×10^{-5}	4.48×10^{-3}	1.52×10^{-6}	4.50×10^{-4}	1.17×10^{-5}	3.46×10^{-3}	9.44×10^{-7}	2.80×10^{-4}	7.26×10^{-6}	2.15×10^{-3}	3.85×10^{-4}	1.63×10^{-3}	2.96×10^{-3}	1.26×10^{-2}
	M	1.90×10^{-6}	5.63×10^{-4}	1.46×10^{-5}	4.33×10^{-3}	1.49×10^{-6}	4.42×10^{-4}	1.15×10^{-5}	3.40×10^{-3}	9.63×10^{-7}	2.86×10^{-4}	7.41×10^{-6}	2.20×10^{-3}	3.33×10^{-4}	1.41×10^{-3}	2.56×10^{-3}	1.09×10^{-2}
**Co**	F	6.83×10^{-7}	4.05×10^{-5}	1.68×10^{-5}	9.94×10^{-4}	5.28×10^{-7}	3.13×10^{-5}	1.30×10^{-5}	7.68×10^{-4}	3.28×10^{-7}	1.95×10^{-5}	8.06×10^{-6}	4.78×10^{-4}	1.34×10^{-4}	1.14×10^{-4}	3.29×10^{-3}	2.79×10^{-3}
	M	6.60×10^{-7}	3.91×10^{-5}	1.62×10^{-5}	9.61×10^{-4}	5.19×10^{-7}	3.08×10^{-5}	1.27×10^{-5}	7.55×10^{-4}	3.35×10^{-7}	1.99×10^{-5}	8.22×10^{-6}	4.88×10^{-4}	1.16×10^{-4}	9.83×10^{-5}	2.84×10^{-3}	2.41×10^{-3}
**CrIII**	F	1.79×10^{-7}	1.38×10^{-5}	3.40×10^{-6}	2.62×10^{-4}	1.38×10^{-7}	1.06×10^{-5}	2.63×10^{-6}	2.03×10^{-4}	8.59×10^{-8}	6.62×10^{-6}	1.64×10^{-6}	1.26×10^{-4}	3.50×10^{-5}	3.87×10^{-5}	6.67×10^{-4}	7.36×10^{-4}
	M	1.73×10^{-7}	1.33×10^{-5}	3.29×10^{-6}	2.54×10^{-4}	1.36×10^{-7}	1.05×10^{-5}	2.58×10^{-6}	1.99×10^{-4}	8.76×10^{-8}	6.76×10^{-6}	1.67×10^{-6}	1.29×10^{-4}	3.03×10^{-5}	3.34×10^{-5}	5.77×10^{-4}	6.37×10^{-4}
**CrVI**	F	1.24×10^{-3}	4.59×10^{-3}	2.36×10^{-2}	8.74×10^{-2}	9.57×10^{-4}	3.55×10^{-3}	1.82×10^{-2}	6.76×10^{-2}	5.95×10^{-4}	2.21×10^{-3}	1.13×10^{-2}	4.20×10^{-2}	2.43×10^{-1}	1.29×10^{-2}	4.62×10^{0}	2.45×10^{-1}
	M	1.20×10^{-3}	4.44×10^{-3}	2.28×10^{-2}	8.45×10^{-2}	9.41×10^{-4}	3.49×10^{-3}	1.79×10^{-2}	6.64×10^{-2}	6.07×10^{-4}	2.25×10^{-3}	1.16×10^{-2}	4.29×10^{-2}	2.10×10^{-1}	1.11×10^{-2}	4.00×10^{0}	2.12×10^{-1}
**Cu**	F	1.23×10^{-3}	7.00×10^{-2}	8.70×10^{-5}	5.16×10^{-3}	9.53×10^{-4}	5.41×10^{-2}	6.72×10^{-5}	3.99×10^{-3}	5.93×10^{-4}	3.37×10^{-2}	4.18×10^{-5}	2.48×10^{-3}	2.09×10^{-1}	1.70×10^{-1}	2.42×10^{-1}	1.97×10^{-1}
	M	1.19×10^{-3}	6.77×10^{-2}	8.41×10^{-5}	4.99×10^{-3}	9.37×10^{-4}	5.32×10^{-2}	6.61×10^{-5}	3.92×10^{-3}	6.05×10^{-4}	3.44×10^{-2}	4.27×10^{-5}	2.53×10^{-3}	1.47×10^{-2}	1.25×10^{-2}	1.71×10^{-2}	1.45×10^{-2}
**Ni**	F	1.29×10^{-6}	3.06×10^{-4}	1.38×10^{-5}	3.27×10^{-3}	9.96×10^{-7}	2.36×10^{-4}	1.07×10^{-5}	2.53×10^{-3}	6.20×10^{-7}	1.47×10^{-4}	6.63×10^{-6}	1.57×10^{-3}	2.53×10^{-4}	8.58×10^{-4}	2.70×10^{-3}	9.18×10^{-3}
	M	1.25×10^{-6}	2.96×10^{-4}	1.33×10^{-5}	3.16×10^{-3}	9.79×10^{-7}	2.32×10^{-4}	1.05×10^{-5}	2.48×10^{-3}	6.32×10^{-7}	1.50×10^{-4}	6.76×10^{-6}	1.60×10^{-3}	2.18×10^{-4}	7.42×10^{-4}	2.34×10^{-3}	7.94×10^{-3}
**Sb**	F	4.31×10^{-6}	3.84×10^{-3}	1.11×10^{-6}	9.83×10^{-4}	3.33×10^{-6}	2.97×10^{-3}	8.54×10^{-7}	7.60×10^{-4}	2.07×10^{-6}	1.85×10^{-3}	5.31×10^{-7}	4.73×10^{-4}	8.46×10^{-4}	1.08×10^{-2}	2.17×10^{-4}	2.76×10^{-3}
	M	4.17×10^{-6}	3.71×10^{-3}	1.07×10^{-6}	9.50×10^{-4}	3.28×10^{-6}	2.92×10^{-3}	8.39×10^{-7}	7.47×10^{-4}	2.12×10^{-6}	1.88×10^{-3}	5.42×10^{-7}	4.82×10^{-4}	7.31×10^{-4}	9.32×10^{-3}	1.87×10^{-4}	2.39×10^{-3}
**Sn**	F	1.40×10^{-7}	8.30×10^{-6}	3.14×10^{-7}	1.86×10^{-5}	1.08×10^{-7}	6.42×10^{-6}	2.43×10^{-7}	1.44×10^{-5}	6.73×10^{-8}	3.99×10^{-6}	1.51×10^{-7}	8.96×10^{-6}	2.74×10^{-5}	2.33×10^{-5}	6.16×10^{-5}	5.23×10^{-5}
	M	1.35×10^{-7}	8.03×10^{-6}	3.04×10^{-7}	1.80×10^{-5}	1.06×10^{-7}	6.31×10^{-6}	2.39×10^{-7}	1.41×10^{-5}	6.87×10^{-8}	4.07×10^{-6}	1.54×10^{-7}	9.14×10^{-6}	2.37×10^{-5}	2.02×10^{-5}	5.32×10^{-5}	4.52×10^{-5}
**Sr**	F	4.97×10^{-5}	2.95×10^{-3}	2.60×10^{-6}	1.54×10^{-4}	3.84×10^{-5}	2.28×10^{-3}	2.01×10^{-6}	1.19×10^{-4}	2.39×10^{-5}	1.42×10^{-3}	1.25×10^{-6}	7.41×10^{-5}	9.74×10^{-3}	8.28×10^{-3}	5.09×10^{-4}	4.33×10^{-4}
	M	4.80×10^{-5}	2.85×10^{-3}	2.51×10^{-6}	1.49×10^{-4}	3.78×10^{-5}	2.24×10^{-3}	1.97×10^{-6}	1.17×10^{-4}	2.44×10^{-5}	1.45×10^{-3}	1.27×10^{-6}	7.56×10^{-5}	8.43×10^{-3}	7.16×10^{-3}	4.41×10^{-4}	3.74×10^{-4}

Table S4. Cancer risk (CR) for all Potentially Toxic Elements (PTEs) determined in face paints and pancakes, results for dermal and ingestion exposures for both sexes in children and adults.

CR		**Children: age 2 to < 3 years**				**Children: age 3 to < 6 years**				**Children: age 6 to < 11 years**				**Adults: age ≥ 21 years**			
		Face paints		Pancake		Face paints		Pancake		Face paints		Pancake		Face paints		Pancake	
**PTEs**	**Sex**	Dermal	Ingestion	Dermal	Ingestion	Dermal	Ingestion	Dermal	Ingestion	Dermal	Ingestion	Dermal	Ingestion	Dermal	Ingestion	Dermal	Ingestion
**As**	F	2.88×10^{-7}	3.98×10^{-5}	6.35×10^{-7}	8.79×10^{-5}	6.67×10^{-7}	3.08×10^{-5}	1.47×10^{-6}	6.79×10^{-5}	6.92×10^{-7}	1.92×10^{-5}	1.53×10^{-6}	4.23×10^{-5}	1.97×10^{-3}	1.12×10^{-4}	3.77×10^{-3}	2.47×10^{-4}
	M	2.78×10^{-7}	3.85×10^{-5}	6.14×10^{-7}	8.50×10^{-5}	6.56×10^{-7}	3.03×10^{-5}	1.45×10^{-6}	6.68×10^{-5}	7.06×10^{-7}	1.95×10^{-5}	1.56×10^{-6}	4.31×10^{-5}	1.71×10^{-3}	9.67×10^{-5}	4.36×10^{-3}	2.14×10^{-4}
**CrVI**	F	2.65×10^{-8}	6.89×10^{-6}	5.05×10^{-7}	1.31×10^{-4}	6.15×10^{-8}	5.32×10^{-6}	1.17×10^{-6}	1.01×10^{-4}	6.38×10^{-8}	3.31×10^{-6}	1.22×10^{-6}	6.31×10^{-5}	4.55×10^{-4}	1.93×10^{-5}	3.00×10^{-3}	3.68×10^{-4}
	M	2.56×10^{-8}	6.66×10^{-6}	4.89×10^{-7}	1.27×10^{-4}	6.05×10^{-8}	5.23×10^{-6}	1.15×10^{-6}	9.96×10^{-5}	6.51×10^{-8}	3.38×10^{-6}	1.24×10^{-6}	6.43×10^{-5}	3.94×10^{-4}	1.67×10^{-5}	8.67×10^{-3}	3.18×10^{-4}
**Pb**	F	1.90×10^{-11}	7.88×10^{-8}	1.79×10^{-10}	7.44×10^{-7}	4.40×10^{-11}	6.09×10^{-8}	4.15×10^{-10}	5.75×10^{-7}	4.56×10^{-11}	3.79×10^{-8}	4.31×10^{-10}	3.58×10^{-7}	1.30×10^{-7}	2.21×10^{-7}	1.06×10^{-6}	2.09×10^{-6}
	M	1.83×10^{-11}	7.62×10^{-8}	1.73×10^{-10}	7.19×10^{-7}	4.32×10^{-11}	5.99×10^{-8}	4.08×10^{-10}	5.65×10^{-7}	4.65×10^{-11}	3.87×10^{-8}	4.40×10^{-10}	3.65×10^{-7}	1.13×10^{-7}	1.91×10^{-7}	1.23×10^{-6}	1.81×10^{-6}
